# Loss of the Sympathetic Signal Produces Sterile Inflammation of the Prostate

**DOI:** 10.3389/fnmol.2022.855376

**Published:** 2022-05-10

**Authors:** Hao Hu, Yiwen Cui, Jing Yang, Ying Cao

**Affiliations:** ^1^Department of Urology, Peking University People’s Hospital, Beijing, China; ^2^Peking University-Tsinghua University-National Institute of Biological Sciences Joint Graduate Program, Peking University, Beijing, China; ^3^Academy for Advanced Interdisciplinary Studies, Peking University, Beijing, China; ^4^Shenzhen Bay Laboratory, Shenzhen, China; ^5^Center for Life Sciences, Peking University, Beijing, China

**Keywords:** 3D fluorescence imaging, prostate, sympathetic innervations, β2-adrenergic receptor, chronic non-bacterial prostatitis

## Abstract

Neural innervations exert essential roles in the prostate. However, spatial distribution and regulatory function of such neural inputs are incompletely characterized. Here, we exploited the advanced whole-tissue immunolabeling and optical clearing technique to assess the 3D anatomy of autonomic innervations in the mouse and human prostate for the first time. We observed that sympathetic and parasympathetic inputs in the mouse prostate remained unaffected during castration-induced tissue regression. However, the pharmacologic destruction of sympathetic innervations in the mouse prostate led to sterile inflammation of the tissue, mimicking the disease condition of chronic non-bacterial prostatitis. Also, the genetic ablation of sympathetic inputs produced a similar inflammatory response. Furthermore, we showed that treatment of the specific β2-adrenergic receptor agonists could effectively mitigate the prostate inflammation caused by such sympathetic loss. Together, these results have elucidated the new immunomodulatory function of the sympathetic signal *via* the β2-adrenergic receptor in prostate inflammatory disease.

## Introduction

Neural innervations within the prostate have been documented for decades (Gorg and Werner, 1966; Kato et al., 1971; Shirai et al., 1973; Gosling and Thompson, 1977; Pennefather et al., 2000; Park et al., 2013). It has been well-recognized that autonomic neural signals, i.e., sympathetic and parasympathetic innervations, are essential for prostate contraction to expel prostatic fluid during ejaculation, which is the organ’s central function (Smith and Lebeaux, 1970; Shima, 1973; Pennefather et al., 2000; Ventura et al., 2002; Puppo and Puppo, 2016). In addition, studies have revealed that neural innervations participate in the prostate tissue homeostasis and disease process. For instance, prostate growth would be restricted by the loss of autonomic neural signals (McVary et al., 1994). Also, sympathetic and parasympathetic innervations could critically influence the onset and progression of prostate cancer (Magnon et al., 2013). However, whether autonomic neural signals might exert additional functions in the prostate, e.g., immunomodulation, remains to be investigated.

Most of the reported immunohistochemical results on the neural anatomy of the prostate relied on tissue sections (Gorg and Werner, 1966; Kato et al., 1971; Shirai et al., 1973; Gosling and Thompson, 1977; Sung et al., 2010; Ganzer et al., 2012). Such conventional methods had intrinsic limitations to visualize the 3D neural distribution, precluding the comprehensive assessment on the whole-tissue level. Notably, several strategies of advanced imaging techniques have emerged in the past few years that enable the 3D assessment of various cellular structures in different unsectioned organs (Chung et al., 2013; Susaki et al., 2014; Renier et al., 2016). Despite such technical advances, 3D imaging of neural anatomy in the rodent and human prostate has yet to be attempted.

Chronic non-bacterial prostatitis, which is also referred to as chronic pelvic pain syndrome (CPPS), has a high incidence among prostate diseases and strikes millions of men globally (Habermacher et al., 2006; Anothaisintawee et al., 2011; Murphy et al., 2014). Patients would suffer long-lasting prostate inflammation without detectable pathogenic infection, and as a result, the symptoms could not be alleviated by antibiotic treatments. Studies have shown the up-regulation of pro-inflammatory cytokines, including TNFα, IL-6, and IL-1β, in CPPS patients (John et al., 2001; Orhan et al., 2001). Also, pro-inflammatory chemokines CCL2 and CCL3 have been indicated in the progression of this disease (Thumbikat et al., 2010). However, the exact cause of chronic non-bacterial prostatitis/CPPS is poorly understood. Although several reports implicated abnormalities of neural signals in the disease condition (Lee et al., 2001; Miller et al., 2002; Yilmaz et al., 2007), the underlying mechanism has been uncharted. In particular, how the sympathetic signal might modulate tissue immunity of the prostate remains to be determined.

In this study, we exploited the advanced whole-tissue immunolabeling and optical clearing technique to examine the 3D anatomy of autonomic innervations in the mouse and human prostate for the first time. We observed that sympathetic and parasympathetic inputs in the mouse prostate were unaffected during castration-induced tissue regression. Importantly, the pharmacologic destruction of sympathetic innervations in the mouse prostate led to sterile inflammation of the tissue irresponsive to antibiotics, mimicking the condition of chronic non-bacterial prostatitis/CPPS. Also, the genetic ablation of sympathetic inputs produced a similar inflammation in the mouse prostate. Furthermore, we showed that the specific β2-adrenergic receptor agonists could effectively mitigate the prostate inflammation caused by such sympathetic loss. Together, our results have elucidated the key immunomodulatory function of the sympathetic signal *via* the β2-adrenergic receptor in the prostate, suggesting the potential mechanism of and novel therapeutic entry points for chronic non-bacterial prostatitis/CPPS.

## Materials and Methods

Requests for further information should be directed to the corresponding author Ying Cao (//caoying@pku.edu.cn).

### Whole-Tissue Immunolabeling of Prostate Tissues

The unsectioned mouse or human prostate is processed for the whole-tissue immunolabeling based on the reported iDISCO+ protocol (Renier et al., 2016). Additional optimizations are included as we recently published (Cao and Yang, 2020). The fresh tissues were collected and fixed in 1% paraformaldehyde/10% sucrose/phosphate-buffered saline at room temperature for 60 min and further fixed in 1% paraformaldehyde/phosphate-buffered saline for 240 min. After the fixation, the tissues were washed with phosphate-buffered saline at room temperature for 60 min three times. The tissues were processed through a methanol gradient (MeOH diluted in ddH_2_O) at room temperature: 20% for 120 min, 40% for 120 min, 60% for 120 min, 80% for 120 min, and 100% for 120 min. The tissues were decolorized for 2 days at 4°C with 3% H_2_O_2_ diluted in 100% MeOH. The tissues were then processed through a reverse MeOH gradient at room temperature: 100% for 120 min, 80% for 120 min, 60% for 120 min, 40% for 120 min, and 20% for 120 min. The tissues were permeabilized with 0.1% Deoxycholate/0.2% TritonX-100/10% DMSO/10 mM EDTA/phosphate-buffered saline at room temperature for 24 h.

The tissues were incubated with 0.2% TritonX-100/10% DMSO/phosphate-buffered saline/5% normal donkey serum at room temperature for 24 h. The tissues were then immunolabeled with the primary antibodies (final concentration of 2 μg/ml) in 0.1% Tween-20/5% normal donkey serum/phosphate-buffered saline at room temperature for 3 days. The primary antibodies for the whole-tissue immunolabeling included rabbit anti-PGP9.5 (Proteintech, 14730-1-AP), rat anti-PECAM1 (BD Biosciences, 553370), goat anti-VEGFR3 (R&D Systems, AF743), rabbit anti-TH (Millipore, AB152), and goat anti-VAChT (Millipore, ABN100). The tissues were washed with 0.1% Tween-20/10 μg/ml heparin/phosphate-buffered saline at room temperature for 6 h four times. The tissues were then immunolabeled with the Alexa Fluor dye-conjugated secondary antibodies (Thermo Fisher Scientific, final concentration of 4 μg/ml) in 0.1% Tween-20/5% normal donkey serum/phosphate-buffered saline at room temperature for 3 days. The tissues were washed with 0.1% Tween-20/10 μg/ml heparin/phosphate-buffered saline at 37°C for 8 h six times.

### Optical Clearing of Prostate Tissues

Before the optical-clearing steps, the immunolabeled prostate tissues were made into 0.8% agarose blocks. The tissue blocks were processed through a MeOH gradient at room temperature: 20% for 60 min three times, 40% for 120 min, 60% for 120 min, 80% for 120 min, 100% for 120 min twice, and 100% for 12 h. The tissue blocks were delipidated at room temperature with 50% dichloromethane/50% MeOH for 60 min three times, followed by 100% dichloromethane for 60 min three times. The tissue blocks were optically cleared at room temperature with 100% dibenzyl ether for 12 h twice.

### Lightsheet Imaging of Prostate Tissues

After the immunolabeling and optical clearing, the prostate tissues were scanned by the LaVision Biotec Ultramicroscope II with a 2x/NA0.5 objective. For imaging at high magnification (1.26x), each tissue was scanned with the 4-μm *z*-step by three lightsheets illuminating from the left side. For imaging at low magnification (12.6x), each tissue was scanned with the 1-μm *z*-step by a single lightsheet illuminating from the left side.

The image stacks obtained from the lightsheet imaging were 3D reconstructed by Imaris.^1^ For the display purpose in the figures and movies, a gamma correction (1.3∼1.6) was applied. The representative 3D images in the figures were produced by orthogonal projection. The movies were filmed with 30 frames per second.

### Human Prostate Tissues

Human prostate tissues were collected in compliance with the protocol approved by the Institutional Ethics Committee of Peking University People’s Hospital, and informed consent was signed by each patient. The normal prostate tissues were sampled from three male patients during the transurethral resection of prostate cancer.

### Mouse Procedures

The mouse experiments were conducted in compliance with the protocol approved by the Institutional Animal Care and Use Committee of Peking University.

C57BL/6 mice were purchased from Charles River International. *Th-Cre* (008601) and *TrkA^fl/fl^* (022362) mice were obtained from Jackson Laboratory and in-house bred to generate *Th-Cre; TrkA*^fl/fl^** and control *Th-Cre; TrkA^+/+^* littermates. Mice were housed on the 12 h/12 h light cycle, and a chow diet and water were provided *ad libitum*. Male mice of 8 weeks old were utilized in the experiments unless otherwise specified.

For the castration procedure, mice were anesthetized with 3% isoflurane. A midline incision was made along the skin region of the scrotum. The testes, vas deferens, and the attached connective tissues on both sides were pulled out. After ligating the blood vessels supplying each testis with a 5/0 suture thread, the testes on both sides were removed. The skin incision was then closed by wound clips. The sham surgery was performed with all the steps except the ligation and removal of testes.

For the 6-OHDA treatment, each mouse was intraperitoneally injected with 2 mg 6-OHDA (Sigma, prepared in 200 μl 0.1% ascorbic acid/phosphate-buffered saline). For the antibiotic treatment, each mouse was administered daily with a combination of ampicillin, neomycin, vancomycin, and metronidazole (together prepared in 200 μl sterile saline) at 10 mg/kg of body weight *via* intraperitoneal injection for 5 days.

For the treatment of the α1-adrenergic receptor antagonist, each mouse was administered daily with prazosin (prepared in 200 μl sterile saline) at 5 mg/kg of body weight *via* intraperitoneal injection for 14 days. For the treatment of the α1-adrenergic or β2-adrenergic receptor agonists, each mouse was administered daily with phenylephrine, clenbuterol, or formoterol (prepared in 200 μl sterile saline) at 5 mg/kg of body weight *via* intraperitoneal injection for 5 days.

### Mouse Tissue Analyses

Mouse anterior and ventral prostate lobes were utilized for analyses due to their relatively larger tissue size compared to the lateral or dorsal lobes. For the qPCR analysis of mRNA levels, both sides of the anterior or ventral prostate of each mouse were harvested and pooled. Total RNAs were purified using the RNeasy Mini Kit (Qiagen). mRNA levels were analyzed by the SYBR Green Real-Time PCR Kit (Thermo Fisher Scientific), with *Cyclophilin* mRNA levels being used as the internal control.

For the fluorescence-activated cell sorting (FACS) analysis, both sides of the anterior prostate of each mouse were harvested and pooled. The tissues were digested in Hanks’ balanced salt solution (HBSS) containing 3% heat-inactivated fetal bovine serum/20 μg/ml DNase I/10 μg/ml Liberase (Roche) at 37°C for 20 min and then mashed by a 40-μm cell-strainer. The cells were centrifuged at 1,000 *g* for 10 min and re-suspended in HBSS containing 3% heat-inactivated fetal bovine serum. The cells were stained by FACS antibodies and analyzed on the BD LSRFortessa. In addition, the FACS-stained CD45^+^CD11b^+^F4/80^+^ macrophages were collected on the BD FACSAria for the qPCR analysis of mRNA levels.

For the enzyme-linked immunosorbent assay (ELISA), both sides of the anterior prostate of five saline-treated or 6-OHDA-treated mice were harvested and pooled. The tissues were thoroughly homogenized in 4 ml phosphate-buffered saline containing Protease Inhibitor Cocktail (Roche). A final concentration of 0.1% Tween-20 was then added to the homogenates and incubated at room temperature for 15 min. The homogenates were centrifuged at 20,000 *g* for 10 min to clear the tissue debris. The supernatants were filtered through a 0.22-μm PES filter and analyzed by Proteome Profiler Mouse Cytokine Array (R&D Systems).

### Statistical Methods

Tissue volumes were measured in the reconstructed 3D images by Imaris. For the quantification of neural innervations, four 200 μm × 200 μm × 200 μm volumes were randomly chosen in the reconstructed 3D images, and axons in each volume were manually measured.

All the experiments were repeated at least twice, with consistent results obtained. GraphPad Prism^2^ performed Student’s *t*-test or ANOVA test.

## Results

### 3D Assessment of Neural Innervations in the Mouse and Human Prostate

**FIGURE 1:**
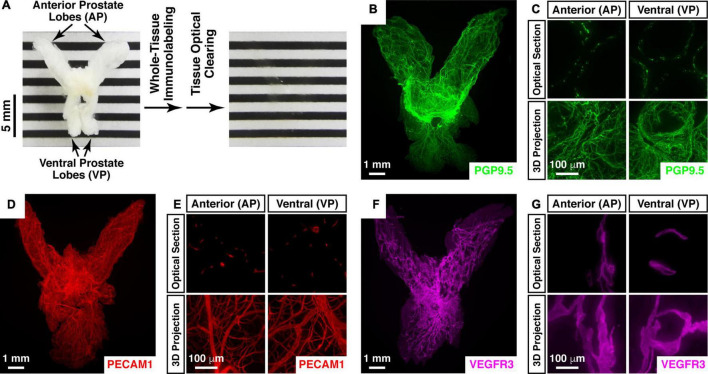
Whole-tissue 3D fluorescence imaging of the mouse prostate. **(A–C)** 3D fluorescence imaging of neural innervations in the mouse prostate. **(A)** The intact prostate of the 8-week-old male mouse before (left panel) and after (right panel) the whole-tissue immunolabeling and optical clearing. Anterior prostate (AP) and ventral prostate lobes (VP) are indicated. **(B,C)** The intact prostate of the 8-week-old male mouse was subjected to the whole-tissue PGP9.5-immunolabeling. **(B)** Representative 3D projection of the lightsheet imaging is shown. **(C)** Representative optical sections (upper panels) and 3D projections (lower panels) of the 1-mm thickness of the anterior (AP) and ventral prostate (VP) of the lightsheet imaging are shown. **(D,E)** 3D assessment of blood vessels in the mouse prostate. The intact prostate of the 8-week-old male mouse was subjected to the whole-tissue PECAM1-immunolabeling. **(D)** Representative 3D projection of the lightsheet imaging is shown. **(E)** Representative optical sections (upper panels) and 3D projections (lower panels) of the 1-mm thickness of the anterior (AP) and ventral prostate (VP) of the lightsheet imaging are shown. **(F,G)** 3D fluorescence imaging of lymphatic vessels in the mouse prostate. The intact prostate of the 8-week-old male mouse was subjected to the whole-tissue VEGFR3-immunolabeling. **(F)** Representative 3D projection of the lightsheet imaging is shown. **(G)** Representative optical sections (upper panels) and 3D projections (lower panels) of the 1-mm thickness of the anterior (AP) and ventral prostate (VP) of the lightsheet imaging are shown.

We aimed at a comprehensive 3D assessment of neural anatomy in the prostate. We pursued the protocol based on the iDISCO+ (immunolabeling-enabled three-dimensional imaging of solvent-cleared organs plus) method (see Section “Materials and Methods”). Importantly, this imaging procedure supports the whole-tissue immunolabeling and optical clearing of prostate tissues. The entire prostate from the adult mouse appeared completely transparent after the processing steps (Figure 1A), thus enabling the lightsheet 3D imaging.

We first subjected the mouse prostate to the whole-tissue immunolabeling of PGP9.5 (protein gene product 9.5), a specific pan-neural marker. The neural architecture in the mouse prostate was revealed for the first time in the field (Figure 1B and Supplementary Video 1). The high-magnification lightsheet optical sections and 3D images showed the neural inputs to the regions such as the anterior and ventral prostate lobes at single-fiber resolution (Figure 1C). Notably, the density of PGP9.5-positive total axons in the ventral prostate (1228 ± 101 mm/mm^3^) was approximately 50% higher than that in the anterior prostate (780 ± 41 mm/mm^3^). As an aside, this 3D imaging technique was also able to visualize different cellular structures in addition to neural innervations. For instance, the whole-tissue immunolabeling of PECAM1 (platelet endothelial cell adhesion molecule 1), a marker for vascular endothelial cells, exhibited the network of blood vessels in the prostate (Figures 1D,E and Supplementary Video 2). Similarly, the whole-tissue immunolabeling of VEGFR3 (vascular endothelial growth factor receptor 3), a marker for lymphatic endothelial cells, demonstrated the 3D distribution of lymphatic vessels in the prostate (Figures 1F,G).

**FIGURE 2:**
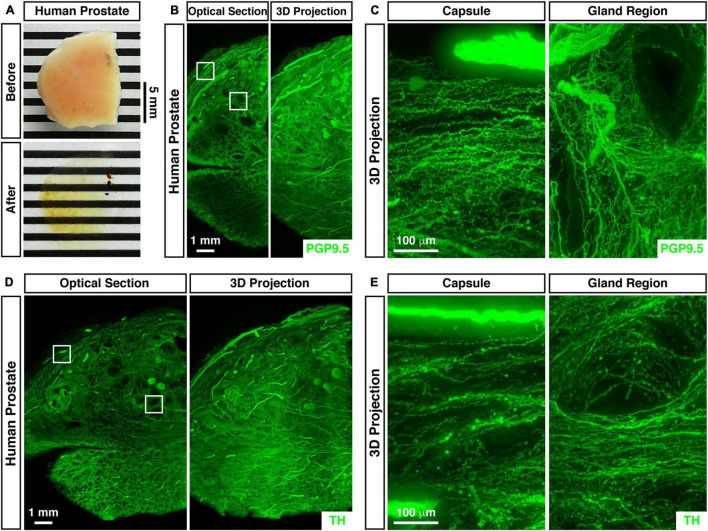
3D assessment of neural innervations in the human prostate. **(A)** The unsectioned prostate tissue of the adult human male before (upper panel) and after (lower panel) the whole-tissue immunolabeling and optical clearing. **(B,C)** 3D fluorescence imaging of neural innervations in the human prostate. The unsectioned prostate tissue of the adult human male was subjected to the whole-tissue PGP9.5-immunolabeling. **(B)** Representative optical section (left panel) and 3D projection (right panel) of the lightsheet imaging are shown. The squares denote the capsule or the gland region imaged at high magnification in panel **(C)**. **(C)** Representative 3D projections of the 1-mm thickness of the capsule (left panel) or the gland region (right panel) of the lightsheet imaging are shown. **(D,E)** 3D assessment of sympathetic innervations in the human prostate. The unsectioned prostate tissue of the adult human male was subjected to the whole-tissue TH-immunolabeling. **(D)** Representative optical section (left panel) and 3D projection (right panel) of the lightsheet imaging are shown. The squares denote the capsule or the gland region imaged at high magnification in panel **(E)**. **(E)** Representative 3D projections of the 1-mm thickness of the capsule (left panel) or the gland region (right panel) of the lightsheet imaging are shown.

We further analyzed the 3D neural distribution of the human prostate. The unsectioned prostate tissue from the adult human male was rendered nearly transparent by the advanced imaging procedure (Figure 2A). The whole-tissue PGP9.5-immunolabeling revealed dense neural innervations throughout the human prostate (Figure 2B). Interestingly, neural innervations beneath the prostate capsule projected parallel while those within the gland region were organized around the acini (Figure 2C). Also, the whole-tissue immunolabeling of TH (tyrosine hydroxylase), a marker for sympathetic innervations, showed the abundant presence of sympathetic distributions within the human prostate (Figure 2D). TH-positive sympathetic axons also exhibited the parallel distribution beneath the capsule but the concentric pattern in the gland region (Figure 2E).

**FIGURE 3:**
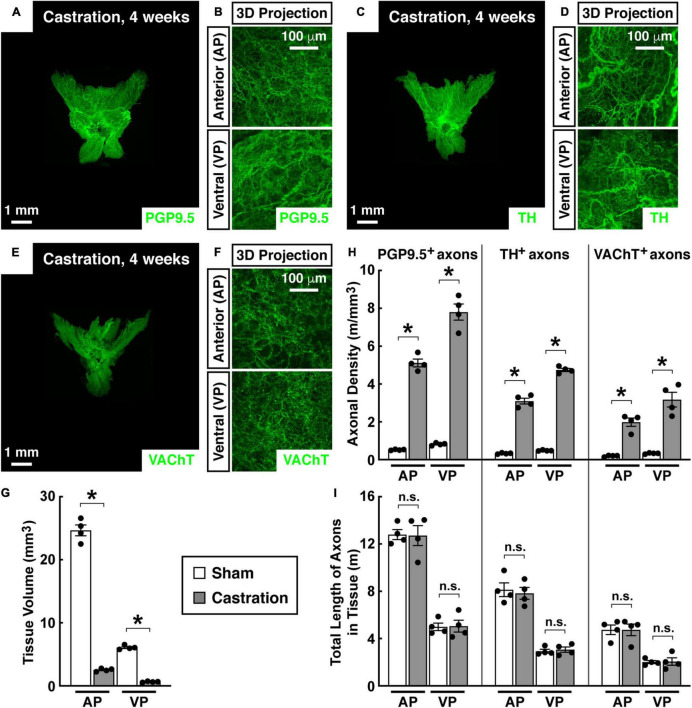
Autonomic innervations were unaffected in the mouse prostate after castration. Eight-week-old male mice were castrated. Four weeks after castration, the intact prostates were harvested and subjected to the whole-tissue immunolabeling of PGP9.5 **(A,B)**, TH **(C,D)**, or VAChT **(E,F)**. **(A,C,E)** Representative 3D projections of the lightsheet imaging are shown. **(B,D,F)** Representative 3D projections of the 1-mm thickness of the anterior (AP) and ventral prostate (VP) of the lightsheet imaging are shown. **(G)** Tissue volumes of the anterior (AP) and ventral prostate (VP) of the mice that underwent castration or sham surgery were quantified. *n* = 4, mean ± SEM, *p < 0.05 (two-way ANOVA test). **(H)** The density of PGP9.5-positive total axons, TH-positive sympathetic axons, or VAChT-positive parasympathetic axons in the anterior (AP) and ventral prostate (VP) was quantified. *n* = 4, mean ± SEM, *p < 0.05 (two-way ANOVA test). **(I)** The total length of PGP9.5-positive axons, TH-positive sympathetic axons, or VAChT-positive parasympathetic axons in the anterior (AP) and ventral prostate (VP) was calculated. *n* = 4, mean ± SEM, n.s., not significant (two-way ANOVA test).

We examined the status of neural innervations in the mouse prostate after castration, a procedure known to induce significant tissue regression. The tissue volumes of the anterior and ventral prostate of the mice receiving sham surgery were 24.67 ± 1.72 mm^3^ and 6.08 ± 0.33 mm^3^, respectively. 4 weeks after castration, the tissue volumes of the anterior (2.53 ± 0.24 mm^3^) and ventral prostate (0.64 ± 0.08 mm^3^) decreased by approximately 90% (Figure 3G). However, the whole-tissue immunolabeling of PGP9.5 suggested a dramatic increase in axonal density within the prostate (Figures 3A,B). In particular, PGP9.5-positive total axons in the anterior prostate were 780 ± 41 mm/mm^3^ in the sham mice but became 7671 ± 619 mm/mm^3^ in the castrated mice. Also, PGP9.5-positive total axons in the ventral prostate increased from 1228 ± 101 mm/mm^3^ in the sham mice to 11703 ± 1104 mm/mm^3^ in the castrated mice (Figure 3H). As a result, the total length of PGP9.5-positive axons calculated in the anterior and ventral prostate appeared unaffected between the sham and castration conditions (Figure 3I). Moreover, the whole-tissue TH-immunolabeling showed a similar increased density of sympathetic innervations in the prostate after castration (Figures 3C,D,H) and the stability of sympathetic innervations after castration (Figure 3I). At the same time, the whole-tissue immunolabeling of VAChT (anti-vesicular acetylcholine transporter), a specific parasympathetic marker, also demonstrated the increase of parasympathetic inputs to the prostate during castration-induced tissue regression (Figures 3E,F,H). Accordingly, the total length of VAChT-positive parasympathetic innervations was comparable between the sham and castration conditions (Figure 3I).

### Loss of Sympathetic Inputs Causes Sterile Inflammation in the Prostate

**FIGURE 4:**
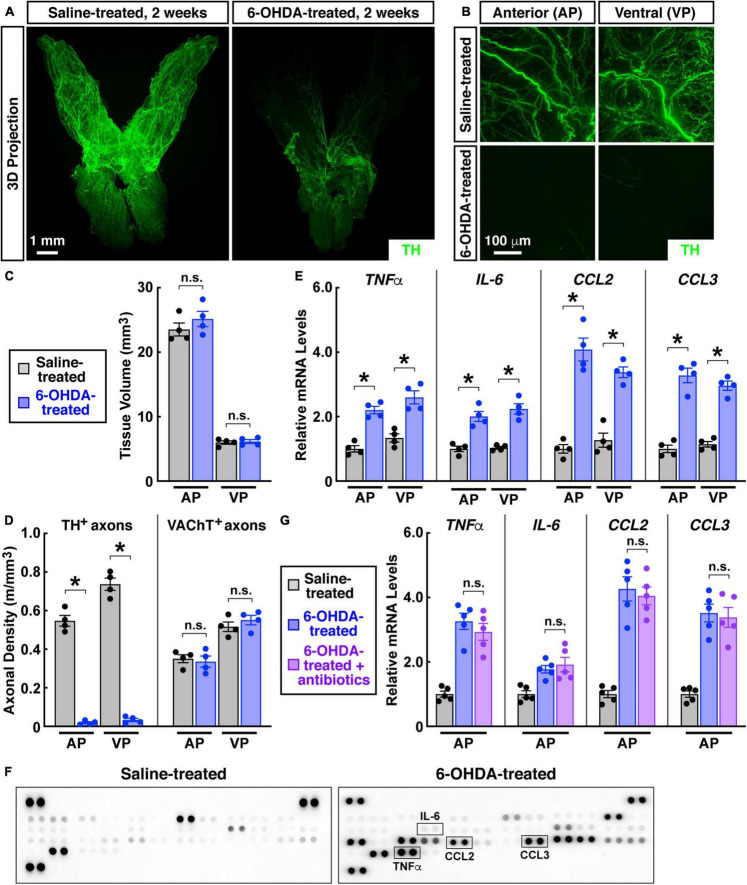
Pharmacologic destruction of sympathetic innervations in the mouse prostate caused sterile inflammation. Eight-week-old male mice were intraperitoneally treated with 6-hydroxydopamine or saline, and the prostates were harvested 2 weeks after the treatment. **(A,B)** The intact prostates were subjected to the whole-tissue TH-immunolabeling. **(A)** Representative 3D projections of the lightsheet imaging are shown. **(B)** Representative 3D projections of the 1-mm thickness of the anterior (AP) and ventral prostate (VP) of the lightsheet imaging are shown. **(C)** Tissue volumes of the anterior (AP) and ventral prostate (VP) of the saline-treated or 6-OHDA-treated mice were quantified. *n* = 4, mean ± SEM, n.s., not significant (two-way ANOVA test). **(D)** TH-positive sympathetic axons or VAChT-positive parasympathetic axons in the anterior (AP) and ventral prostate (VP) were quantified. *n* = 4, mean ± SEM, *p < 0.05, n.s., not significant (two-way ANOVA test). **(E)** mRNA levels of pro-inflammatory cytokines and chemokines in the anterior (AP) and ventral prostate (VP) were analyzed by the qPCR analysis. *n* = 4, mean ± SEM, *p < 0.05 (two-way ANOVA test). **(F)** Cytokines and chemokines in the anterior prostate were analyzed by the mouse cytokine array. **(G)** The mice at 2 weeks after the 6-OHDA treatment were daily treated by antibiotics *via* intraperitoneal injection for 5 days. mRNA levels of pro-inflammatory cytokines and chemokines in the anterior prostate (AP) were determined by the qPCR analysis. *n* = 5, mean ± SEM, n.s., not significant (one-way ANOVA test).

Aided by the advanced imaging technique, we focused on the sympathetic innervations in the prostate. We first exploited the pharmacologic sympathetic ablation by the intraperitoneal treatment of 6-OHDA (6-hydroxydopamine). The whole-tissue TH-immunolabeling and 3D imaging exhibited the effective removal of sympathetic innervations in the prostate 2 weeks after the 6-OHDA treatment (Figures 4A,B). TH-positive sympathetic inputs to the anterior prostate were 547 ± 56 mm/mm^3^ in the saline-treated control but became 19 ± 10 mm/mm^3^ in the 6-OHDA-treated condition. Sympathetic innervations in the ventral prostate also decreased from 737 ± 64 mm/mm^3^ in the control condition to 33 ± 17 mm/mm^3^ after the 6-OHDA treatment (Figure 4D). Notably, such pharmacologic sympathetic ablation did not alter the general anatomy or tissue volume of the prostate (Figure 4C). As expected, the whole-tissue VAChT-immunolabeling showed that parasympathetic innervations were unaffected in the 6-OHDA-treated prostate (Figure 4D).

Of importance, we observed that the pharmacologic destruction of sympathetic innervations led to spontaneous inflammation in the prostate. Significant up-regulation of pro-inflammatory cytokines *TNFa* and *IL-6* occurred in the anterior and ventral prostate (Figure 4E). At the same time, expression levels of pro-inflammatory chemokines *CCL2* and *CCL3* increased in the 6-OHDA-treated prostate (Figure 4E). These pro-inflammatory factors have been indicated in the disease process of chronic non-bacterial prostatitis/CPPS (John et al., 2001; Orhan et al., 2001; Thumbikat et al., 2010). Such increased levels of pro-inflammatory cytokines and chemokines in the 6-OHDA-treated prostate were confirmed by the ELISA on the mouse cytokine array (Figure 4F). We then tested whether this prostate inflammation caused by sympathetic ablation would respond to antibiotics. The combinatorial treatment of four antibiotics (i.e., ampicillin, neomycin, vancomycin, and metronidazole) did not mitigate the increased pro-inflammatory cytokines and chemokines in the 6-OHDA-treated prostate (Figure 4G), ruling out the involvement of any bacterial infection due to the intraperitoneal injection procedure. Such irresponsiveness to antibiotics recapitulated another key feature of chronic non-bacterial prostatitis/CPPS.

**FIGURE 5:**
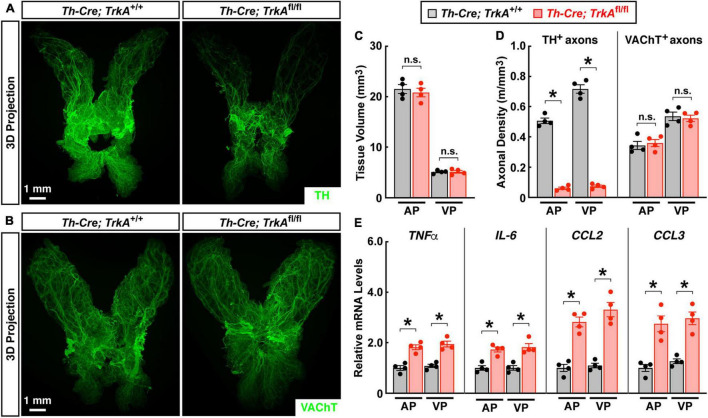
Genetic ablation of sympathetic innervations produced sterile inflammation in the prostate. The prostates of *Th-Cre; TrkA*^fl/fl^** and control *Th-Cre; TrkA^+/+^* mice of 8 weeks old were harvested. **(A,B)** The intact prostates were subjected to the whole-tissue immunolabeling of TH **(A)** or VAChT **(B)**. Representative 3D projections of the lightsheet imaging are shown. **(C)** Tissue volumes of the anterior (AP) and ventral prostate (VP) of *Th-Cre; TrkA*^fl/fl^** and control *Th-Cre; TrkA^+/+^* mice were quantified. *n* = 4, mean ± SEM, n.s., not significant (two-way ANOVA test). **(D)** TH-positive sympathetic axons or VAChT-positive parasympathetic axons in the anterior (AP) and ventral prostate (VP) were quantified. *n* = 4, mean ± SEM, *p < 0.05, n.s., not significant (two-way ANOVA test). **(E)** mRNA levels of pro-inflammatory cytokines and chemokines in the anterior (AP) and ventral prostate (VP) of *Th-Cre; TrkA*^fl/fl^** and control *Th-Cre; TrkA^+/+^* mice were analyzed by the qPCR analysis. *n* = 4, mean ± SEM, *p < 0.05 (two-way ANOVA test).

We went on to validate the observation that loss of sympathetic inputs would produce sterile inflammation in the prostate. TrkA (tyrosine kinase receptor A) is one of the central neurotrophin receptors for establishing sympathetic innervations (Kuruvilla et al., 2004). We found that the specific deletion of *TrkA* in sympathetic neurons in *Th-Cre; TrkA*^fl/fl^** mice resulted in the significant loss of TH-positive sympathetic axons in the prostate (Figures 5A,D). The lateral and dorsal prostate lobes seemed less sensitive to such sympathetic ablation in *Th-Cre; TrkA*^fl/fl^** mice, though the underlying reason is not immediately clear. On the other side, VAChT-positive parasympathetic axons were not affected in *Th-Cre; TrkA*^fl/fl^** mice (Figures 5B,D). We noted a prior study reporting that prostate development might be affected by the loss of autonomic neural signals (McVary et al., 1994). However, the general anatomy and tissue volume of the *Th-Cre; TrkA*^fl/fl^** prostate remained normal compared to that in the *Th-Cre; TrkA^+/+^* control prostate (Figure 5C). Importantly, this genetic loss of sympathetic innervations caused a spontaneous inflammation in the *Th-Cre; TrkA*^fl/fl^** prostate, as characterized by the up-regulation of pro-inflammatory cytokines and chemokines (Figure 5E), similar to that triggered by the pharmacologic sympathetic ablation.

### β2-Adrenergic Receptor Signaling Inhibits Prostate Inflammation

**FIGURE 6:**
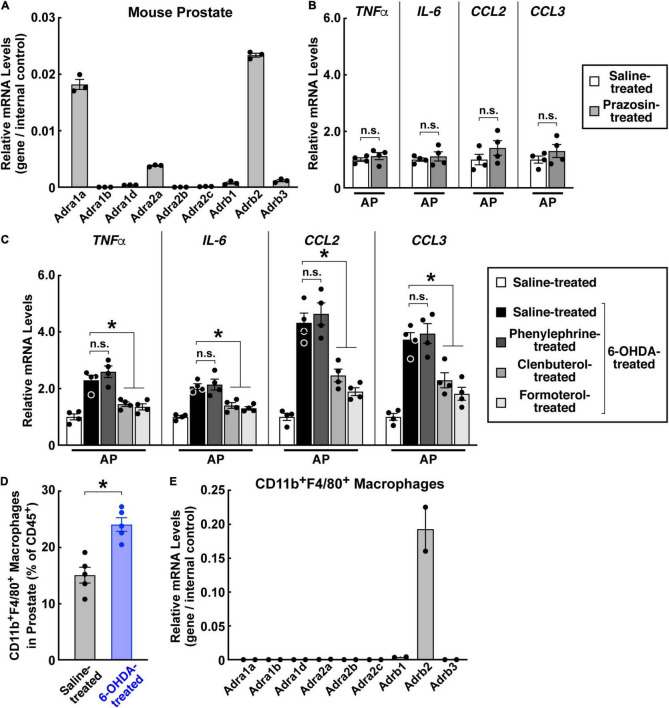
β2-Adrenergic receptor signal suppressed the prostate inflammation caused by sympathetic loss. **(A)** Expression profile of adrenergic receptors in the mouse prostate. mRNA levels of adrenergic receptors in the prostates of 8-week-old male mice were analyzed by the qPCR analysis. *n* = 3, mean ± SEM. **(B)** The α1-adrenergic receptor antagonist did not cause prostate inflammation. Eight-week-old male mice were daily administered with prazosin *via* intraperitoneal injection for 14 days. mRNA levels of pro-inflammatory cytokines and chemokines in the anterior prostate (AP) were analyzed by the qPCR analysis. *n* = 4, mean ± SEM, n.s., not significant (Student’s *t*-test). **(C,D)** 8-week-old male mice were intraperitoneally treated with 6-hydroxydopamine or saline control. **(C)** The β2-adrenergic receptor agonists suppressed prostate inflammation caused by sympathetic loss. The mice at 2 weeks after the 6-OHDA treatment were daily administered with the α1-adrenergic receptor agonist phenylephrine or β2-adrenergic receptor agonists clenbuterol or formoterol *via* intraperitoneal injection for 5 days. mRNA levels of pro-inflammatory cytokines and chemokines in the anterior prostate (AP) were analyzed by the qPCR analysis. *n* = 4, mean ± SEM, *p < 0.05, n.s., not significant (one-way ANOVA test). **(D)** Increased accumulation of CD11b^+^F4/80^+^ macrophages during prostate inflammation. Immune cells in the anterior prostate at 2 weeks after the 6-OHDA treatment were examined by the FACS analysis. *n* = 5, mean ± SEM, *p < 0.05 (Student’s *t*-test). **(E)** Expression profile of adrenergic receptors in CD11b^+^F4/80^+^ macrophages of the prostate. mRNA levels of adrenergic receptors in the CD11b^+^F4/80^+^ macrophages FACS-sorted from the prostate were analyzed by the qPCR analysis. *n* = 2, mean.

We then investigated adrenergic signaling downstream of sympathetic inputs in the prostate. Previous studies have mainly focused on the functions of α1-adrenergic receptors in the prostate. In particular, the α1-adrenergic receptor antagonist prazosin has been commonly utilized for treating benign prostatic hyperplasia (Forray et al., 1994; Roehrborn and Schwinn, 2004; Schwinn and Roehrborn, 2008). We confirmed the abundant expression of the α1-adrenergic receptor *Adra1a* in the mouse prostate (Figure 6A). However, unlike that observed above with loss of sympathetic innervations, blockage of the α1-adrenergic receptor signal with prazosin failed to produce the inflammatory condition in the prostate (Figure 6B). In addition, activation of the α1-adrenergic receptor by the specific agonist phenylephrine did not affect the prostate inflammation caused by the sympathetic ablation (Figure 6C). Therefore, we looked into the potential involvement of other adrenergic receptors. The expression profiling showed that the β2-adrenergic receptor *Adrb2* was also highly expressed in the prostate (Figure 6A). More importantly, we demonstrated that treatment of the β2-adrenergic receptor agonists clenbuterol or formoterol was sufficient to suppress the pro-inflammatory cytokines and chemokines in the 6-OHDA-treated prostate (Figure 6C), thus identifying an essential role of β2-adrenergic receptor signaling in this disease context. Finally, we explored the immune cells potentially mediating the sympathetic signal in the prostate. The FACS analysis showed an increased accumulation of CD11b^+^F4/80^+^ macrophages in the 6-OHDA-treated prostate compared to that in the saline-treated control (Figure 6D). Further, these CD11b^+^F4/80^+^ macrophages exhibited a high expression of *Adrb2* (Figure 6E), implicating their direct participation in prostate inflammation.

## Discussion

In this study, we reported the 3D assessment of autonomic innervations in the mouse and human prostate tissues for the first time. Recent studies have begun to reveal the critical involvement of such autonomic inputs in the onset and progression of prostate cancer (Magnon et al., 2013; March et al., 2020; Zahalka and Frenette, 2020). It thus becomes conceivable that the advanced 3D imaging technique could be exploited to determine the neural distribution in clinical samples of human prostate cancer, providing more comprehensive insights into the tumor pathology than conventional immunohistochemistry methods. Also, the 3D imaging technique is readily applicable to additional cellular structures in the prostate, e.g., blood vessels and lymphatic vessels, as we successfully demonstrated.

We elucidated the essential function of sympathetic inputs in the immune regulation of the prostate. In particular, loss of sympathetic innervations in the mouse prostate produced the spontaneous, sterile inflammation characterized by the increased levels of pro-inflammatory cytokines and chemokines and the irresponsiveness to antibiotics. Therefore, such pharmacologic or genetic sympathetic ablation in the mouse prostate could recapitulate some of the key features of chronic non-bacterial prostatitis/CPPS. Of interest, the density of sympathetic innervations in the prostate would increase significantly in the castrated mice. Whether such locally increased sympathetic inputs might affect tissue immunity in the prostate remains to be determined. Notably, experimental autoimmune prostatitis in rodents has been utilized to delineate the pathological mechanisms underlying chronic non-bacterial prostatitis/CPPS (Motrich et al., 2007; Rudick et al., 2008). Our work has established an additional mouse model to explore potential neuroimmune interactions in this complex prostate disease. Furthermore, whether the local sympathetic neuropathy might exert a causative role in chronic non-bacterial prostatitis/CPPS in patients calls for future investigations.

Adrenergic signals in the prostate have been extensively studied. Prior works have shown that the α1-adrenergic receptor is highly expressed in the prostate. Moreover, the α1-adrenergic receptor antagonist prazosin has been broadly prescribed for alleviating the symptoms of benign prostatic hyperplasia (Forray et al., 1994; Roehrborn and Schwinn, 2004; Schwinn and Roehrborn, 2008). However, we observed that prazosin or the α1-adrenergic receptor agonist phenylephrine did not affect the prostate inflammation caused by sympathetic loss, implying the minor involvement of α1-adrenergic receptor signaling in this context. On the other hand, we found that the β2-adrenergic receptor is expressed in the prostate. Importantly, its specific agonists clenbuterol and formoterol could effectively revert the inflammatory condition. This observation has suggested the β2-adrenergic receptor as a novel target for the therapeutic intervention of chronic non-bacterial prostatitis/CPPS. In addition, a recent study reported that the angiogenesis regulated by the β2-adrenergic receptor could participate in prostate cancer (Zahalka et al., 2017). More efforts have been warranted to explore the basic biology and translational research of β2-adrenergic receptor signaling in the prostate.

In sum, our study has elucidated the critical function of the sympathetic signal *via* the β2-adrenergic receptor in modulating prostate inflammation, which would offer valuable insights into the knowledge of the physiology and disease of this gland organ.

## Data Availability Statement

The original contributions presented in the study are included in the article/Supplementary Material, further inquiries can be directed to the corresponding author.

## Ethics Statement

The study involving human participants was reviewed and approved by Institutional Ethics Committee of Peking University People’s Hospital. The patients/participants provided their written informed consent to participate in this study. The animal study was reviewed and approved by Institutional Animal Care and Use Committee (IACUC) of Peking University.

## Author Contributions

HH collected the human prostate tissues. YCa wrote the manuscript. All authors conducted the experiments and the data analyses and approved the submitted version.

## Conflict of Interest

The authors declare that the research was conducted in the absence of any commercial or financial relationships that could be construed as a potential conflict of interest.

## Publisher’s Note

All claims expressed in this article are solely those of the authors and do not necessarily represent those of their affiliated organizations, or those of the publisher, the editors and the reviewers. Any product that may be evaluated in this article, or claim that may be made by its manufacturer, is not guaranteed or endorsed by the publisher.

## Footnotes

1
https://imaris.oxinst.com/packages

2
http://www.graphpad.com/scientific-software/prism

## Supplementary Material

Supplementary Video 1 3D fluorescence imaging of neural innervations in the mouse prostate. The intact prostate of the 8-week-old male mouse was subjected to the whole-tissue PGP9.5-immunolabeling. 3D reconstruction of neural innervations in the prostate is shown.

Supplementary Video 2 3D assessment of blood vessels in the mouse prostate. The intact prostate of the 8-week-old male mouse was subjected to the whole-tissue PECAM1-immunolabeling. 3D reconstruction of blood vessels within the 1-mm thickness of the anterior prostate is shown.
